# The end of life experiences of people living with socio-economic deprivation in the developed world: an integrative review

**DOI:** 10.1186/s12904-022-01080-6

**Published:** 2022-11-05

**Authors:** Sarah P Bowers, Ming Chin, Maire O’Riordan, Emma Carduff

**Affiliations:** 1 NHS Tayside and University of Dundee, Ninewells Hospital, Dundee, DD1 9SY UK; 2 University Hospital Wishaw, 50 Netherton Street, Lanarkshire, ML2 0DP UK; 3 Marie Curie Hospice, 133 Balornock Road, Glasgow, G21 3US UK

**Keywords:** Palliative Care, Terminal Care, Socioeconomic Factors, Social Class, Delivery of Health Care, Integrative review

## Abstract

**Background:**

Those experiencing socioeconomic deprivation have poorer quality of health throughout their life course which can result in poorer quality of death – with decreased access to palliative care services, greater use of acute care, and reduced access to preferred place of care compared with patients from less deprived populations.

**Aim:**

To summarise the current global evidence from developed countries on end-of-life experience for those living with socio-economic deprivation.

**Design:**

Integrative review in accordance with PRISMA*.* A thorough search of major databases from 2010–2020, using clear definitions of end-of-life care and well-established proxy indicators of socio-economic deprivation. Empirical research describing experience of adult patients in the last year of life care were included.

**Results:**

Forty studies were included from a total of 3508 after screening and selection. These were deemed to be of high quality; from a wide range of countries with varying healthcare systems; and encompassed all palliative care settings for patients with malignant and non-malignant diagnoses. Three global themes were identified: 1) multi-dimensional symptom burden, 2) preferences and planning and 3) health and social care interactions at the end of life.

**Conclusions:**

Current models of healthcare services are not meeting the needs of those experiencing socioeconomic deprivation at the end-of-life. Further work is needed to understand the disparity in care, particularly around ensuring patients voices are heard and can influence service development and delivery.

## Introduction

On a global scale, it is now recognized that people experiencing socioeconomic deprivation (SED) are spending more of their shorter lives in ill health and are carrying a higher burden of chronic disease, multimorbidity and symptom burden than more affluent neighbours [1–3]. Socioeconomic deprivation is an independent risk factor for higher mortality in both cancer and non-cancer populations [4–6] and is associated with higher use and cost of healthcare in the last year of life [7, 8].

Given that people living with SED are carrying this inequitably high burden of poor health outcomes, equal access to palliative care would be expected when compared with those in more affluent areas. However, it is now recognised that people with lower incomes have a potentially poorer experience at end-of-life (EOL) [6], with reduced referral and access to specialist palliative care services in and out of hours [9–11], are more likely to use ambulance and A&E and to be admitted to and die in hospital [7, 12, 13], rather than at home or in a hospice [9, 10, 14–17]. People with lower wealth have recently been found to have had more hospital admissions in the last two years of life [18] and this is often a substitute for elective, community-based healthcare services [19]. Families living in poverty may need to compromise on food and heating and incur significant debts when someone dies [20] and report significantly less support and satisfaction with care received at the EOL [6]. A qualitative evidence synthesis, undertaken in 2019, identified that access to preferred place of death was limited by human factors, such as social support, personal and cultural beliefs, poor communication, and environmental factors, such as suitability of the home environment, and availability of resources within health and care services [21]. An earlier review highlighted barriers such as accessibility, availability, affordability and acceptability [22].

As quantitative evidence describing the disparity in access and delivery of palliative care services between socioeconomic groups continues to grow, there is still little understanding of how people with SED experience living and dying with a life-limiting illness. This review feeds directly into ongoing empirical work to understand the experiences of home death for people at the EOL who are living with poverty [23] and complements a recently published paper by Rowley and colleagues [24] by providing a comprehensive and systematic description of the issues experienced at EOL. This manuscript provides further justification for Rowley et al.’s call to action for researchers, policy-makers and clinicians working with people experiencing socioeconomic deprivation at EOL [24]. By understanding what inequalities and disparities persist, practitioners and policy makers can meaningfully address the issue at individual, institutional and societal levels and progress can be made to narrow the gap. The aim of this review is to describe current global evidence from high income countries on the experiences of people at the EOL who are living with SED.

## Methods

### Design

An integrative literature review approach was chosen to facilitate synthesising and concept building given the multiple evidence sources and methodologies. We conducted our review according to the methods described by Whittemore and Knafl [25]. This study was registered on PROSPERO (CRD42019151906) [26] and reported in accordance with Preferred Reporting Items for Systematic Reviews and Meta-Analyses (PRISMA) [27].

### Definitions

We used the NICE definition for EOL which includes people who are likely to die within 12 months, with advanced, progressive, or incurable diseases or acute life-threatening conditions [28]. We also referred to Rietjens recent article to develop our search terms related to palliative care; these were developed from the World Health Organisation broad and accepted definition of palliative care – *“Palliative care is an approach that improves the quality of life of patients and their families facing the problems associated with life-threatening illness, through the prevention and relief of suffering by means of early identification and impeccable assessment and treatment of pain and other problems, physical, psychosocial and spiritual*” [29, 30]. The definitions available for socioeconomic status are less distinct. Clear definitions are rare (i.e. the United Kingdom Index of Multiple Deprivation), do not encompass the complex nature of SED and are particularly poor at capturing rural SED [31]. We therefore reviewed the evidence on proxy indicators of SED in addition to systematic reviews of the same topic to devise the evidence-based list of search terms related to SED which are presented in Table 1 [7, 32, 33]. This review did not include papers from developing countries given their unique challenges to resources and the delivery of healthcare.

**Table 1:** Search Terms

Palliative care	AND	Socio-economic SED
•Palliative care •Palliat* •Terminal care •Terminal* ill* •Terminal diagnos* •Dying •Advanced illness* •Life limit* •EOL •Last year of life •EOLC •Advanced cancer •Advanced progressive illness		•Socioeconomic factor* •Low income •Social class* •Social depriv* •Working class* •Social* disadvantage* •Low education •Uneducated •Social inequal* •Index of depriv* •Socioeconomic status •Poor •Poverty •Occupation

*Allows for searching for multiple endings of the root word

There have been multiple recently published reviews focussing on the interaction of socioeconomic inequities and place of death and access [13, 34–36]. Such studies have utilised different approaches in their definitions of the dynamic concept of access including the Levesque’s five domains [34] and the candidacy model [36]. The similarity in such concepts of access is the identification of patients both by self and professionals as needing a particular healthcare service, the initial contact and subsequent uptake of that service and to actually have a need for the services fulfilled. With this in mind, our criteria (Table 2) excluded papers which solely focused on initial contact with or referral to palliative care services, patient and professional perceptions of palliative care need and availability of resources.

**Table 2:** Inclusion and Exclusion criteria

Inclusion criteria	Exclusion criteria
Articles reporting the experiences of adult patients with a diagnosis of terminal illness who were approaching the EOL from the perspective of the patient or the unpaid/family caregiver	Articles reporting the experiences of those under the age of 18
Articles which described a population of participants who were socio-economically deprived	Articles reporting the experiences of patients living with chronic illness but not thought to be at the EOL
Research was conducted in high income countries (as per World Bank List of economies [37])	Articles reporting the perceptions of health professionals I.e. health professional proxy accounts of experience
Articles were peer reviewed empirical research	Articles which were focused on place of death or on access to palliative care for people at the EOL: initial contact with services, perceptions of services and availability of services
Published in English	Systematic reviews, reports, commentaries, editorials, conference proceedings, case reports, grey literature

### Search strategy

Key search terms were agreed between SB and EC, with support from a librarian based at NHS Greater Glasgow & Clyde, and these were combined with standard Boolean operators (Table 1). The following databases were searched to capture the wide range of research and disciplines involved in SED at the EOL – Medline, Embase, CINAHL, ASSIA and PsychInfo. The most relevant systematic review to describe palliative care in the context of SED was reported in 2010 [22] and thus we limited our search from January 2010 to March 2020. Inclusion and exclusion criteria were agreed to ensure we obtained relevant, original research (Table 2).

### Study selection

Initial screening by title and abstract was conducted by SB and MC. Both authors screened the first 20 papers for inclusion to ensure consistency. Full texts were then reviewed according to the inclusion and exclusion criteria. Where there was uncertainty, papers were read by EC and a decision was made as a team.

### Quality appraisal

Given the breadth of research designs obtained by integrative reviews, there is no gold standard for quality appraisal [25]. However, a research critique framework developed by Caldwell et al. [38] was considered suitable to assess quality in both qualitative and quantitative papers. Bloomer et al. used the Caldwell criteria in a recent integrative review and shared the flowchart they had developed [39]. The methodological strengths and weaknesses of each included study were assessed independently with SB and MC using the Caldwell 11 point criteria. Final scores were corroborated and differences discussed.

### Data abstraction and synthesis

We followed the four steps as described by Whittemore and Knafl: 1) data reduction, 2) data display, 3) data comparison, 4) conclusion drawing [25]. The papers were reviewed and data extracted on: full reference, continent, language, how SED was defined, how EOL was defined, aim, objectives or research questions, design, setting for data collection, participants, diagnosis, sample size, tools, key findings, limitations as described, author conclusions, references to other relevant studies.

With the aim of the review in mind, SB and MC thoroughly read each of the included papers independently and extracted the relevant findings. These were then categorised into 15 broad themes which were assimilated into an excel database. Using the constant comparison method outlined by Whittemore and Knafl [25], extracted data were compared item by item to allowing grouping and categorization allowing varied data from diverse methodology to be collated. The final 3 global themes, and 9 subthemes, were then created based on the above groupings of the findings with corroboration from all authors.

## Findings

### Included studies

**Fig. 1:**
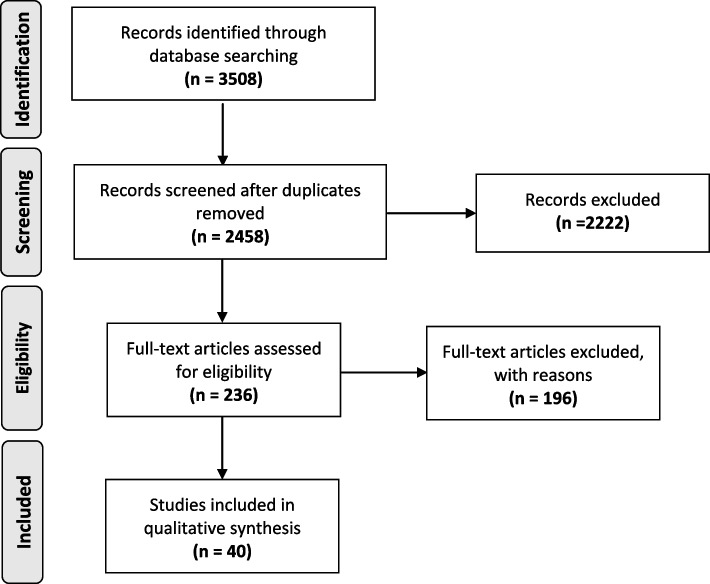
Prisma flow diagram of search results

In total, 2458 studies were found after removal of duplicates as illustrated in Fig. 1. Initial screening of titles and abstracts resulted in 236 papers for full-text review. Ultimately, 40 studies were included in the analysis. A list of these papers is included in Table 3. In total, 38 out of 40 papers were of high quality with a score of 9/11 or higher, the remaining 2 papers scored 8/11. Given the experiences of people living in SED have not been well described in detail in the literature, we did not exclude on the basis of quality.

**Table 3:** Summary of included studies

Author	Aim	Setting (and country)	Sample	Study Design and method	Main Findings	Quality Score
Daugaard et al. (2019) [40]	To examine the association between social and socioeconomic position and drug reimbursement due to terminal illness (DRTI) registration among patients who are terminally ill in Denmark	Population-based (Denmark)	Terminally ill patients who died 2006 – 2015 with cancer or seven other chronic illnesses (*n* = 307,188)	Quantitative Database analysis	-No association between education and DRTI -Higher prevalence of DRTI in middle/high income patients, however palliative care needs may be under-recognised in low income patients -Lower prevalence of DRTI in employed patients	11
Ankuda et al. (2018) [41]	To identify what services are critical and why they matter to patients in a home-based palliative program	Hospice – home based program (US)	Enrollees in the program (*n* = 18)	Mixed Individual interviews and survey	-Participants living in poverty had more non-routine and social work visits and found the social services provided by a home hospice service to be the most critical aspect of care	9
Azhar et al. (2018) [42]	To compare time of referral and characteristics (level of symptom distress) among uninsured (indigent), low-insured (Medicaid), and insured patients presenting to the supportive care center	Outpatient center (US)	Patients with advanced cancer (500)	Quantitative Survey to participants	-Uninsured and low/partially insured patients had higher ESAS pain scores -Indigent patients had: -More encounters with palliative care team per month -Higher levels of distress -Higher risk for aberrant opioid usage and -More frequently on opioids at time of referral to centre -Insurance status did not impact timing of palliative care referral or follow-up	10
Brown et al. (2018) [43]	To examine the association between race/ethnicity, other key social determinants of health, and healthcare intensity in the last 30 days of life for those with chronic, life-limiting illness	Population-based (US)	All patients who died from 1 of 9 chronic conditions between January 2010 and December 2015 (*n* = 22,068)	Quantitative Database analysis	-Lower income was associated with higher likelihood of receiving high intensity care (i.e., treatment in an intensive care unit, receipt of mechanical ventilation, receipt of cardiopulmonary resuscitation) in the last 30 days of life	11
Cervantez et al. (2018) [44]	To evaluate how insurance coverage and ethnicity impact distribution of symptom burden and how those factors influence palliative access for patients at a South Texas cancer center	Outpatient center (US)	Patients who attended clinics (*n* = 604)	Quantitative Survey to participants	-Symptom burden was similar in insured and uninsured patients, but there was a four-fold delay in first palliative care visit in uninsured group	8
Huang et al. (2015) [45]	To test the hypothesis that men and those with lower levels of educational attainment would have less favourable attitudes towards palliative care	Community and hospital-based cancer clinics (US)	Patients with cancer (*n* = 383)	Quantitative Secondary analysis of pre-existing VOICES dataset	-Most patients would desire palliative care if no further anti-cancer treatment would be helpful -No difference in preferences between those with different levels of education	11
Cai et al. (2017) [46]	To examine the socioeconomic differences in the propensity and intensity of health service use for the three main home-based services (physician visits, nurse visits and personal support worker hours), and to explore the determinants of the use of home-based palliative care services	Home-based palliative care program (Canada)	Adult primary caregivers of patients who were diagnosed with malignant neoplasm (*n* = 181)	Quantitative Survey administered to participants	-Patients with lower socioeconomic status were more likely to receive at least one home-based nurse visit or personal support worker (PSW) visit -Lower socioeconomic status was associated with higher intensity of health service use -Socioeconomic status is a significant predictor of propensity and intensity of home-based nurse and PSW visit even after controlling for health status -Patients with higher levels of education were less likely to receive at least one home-based PSW visit	11
Koroukian et al. (2017) [47]	To gain a better understanding of the correlates of aggressive end-of-life care and hospice use in older adults dying from cancer, by accounting for both social determinants of health and multimorbidity	Population-based (US)	Participants < 66 years who died from cancer with complete Medicare claims history (*n* = 835)	Quantitative Database analysis	-Percentage of patients receiving cancer-directed treatment increased with higher education and income -No significant difference by income or education for admission to hospital -Enrolment in hospice was generally higher with higher levels of education or income -There was a U-shaped distribution between in-hospital death and income and education	10
Krishnan et al. (2017) [48]	To examine the family and facility factors that may influence the decision to transfer nursing home residents to hospital in the last month of life	Nursing homes (Canada)	Adult relatives of residents who had died in the past year and was self-reported as the most involved in their care in their last month of life (*n* = 119)	Quantitative Survey to participants	-There was a higher prevalence of hospital transfers among deceased whose family members had lower income -Low income family members were 3 times more likely to send patient to hospital -No statistically significant relationship was noted between employment or education level of the family members and terminal hospital transfers	11
Carlucci et al. (2016) [49]	To elicit the patients’ end-of-life preferences in very severe chronic obstructive pulmonary disease	Hospital (Italy)	Patients with very severe COPD (*n* = 43)	Mixed Individual interview Survey to participants	-Patients with lower education were more likely to choose endotracheal tube intubation -Higher education level is the strongest predictor of probability of choosing palliative care option with oxygen and morphine	9
Dhingra et al. (2016) [50]	To evaluate a diverse population served by an interdisciplinary model of community-based specialist palliative care and the variation in service delivery over time and identify subgroups with distinct illness burden profiles	Community palliative care program (US)	Patients referred to the program (*n* = 894)	Quantitative Survey to participants	-Patients who reported very low illness burden more likely to reside in impoverished neighbourhoods	11
Karikari-Martin et al. (2016) [51]	To examine whether differences in hospice use and use of aggressive services in the last 12 months of life are influenced by race or any cancer diagnosis while adjusting for the covariates age, gender, marital status, education level, neighbourhood socioeconomic status, cognitive function, and physical function	A geographically-defined community in Chicago (US)	Participants < 65 years enrolled Medicare for at least 1 year before death (*n* = 2954)	Quantitative Secondary analysis of pre-existing CHAP data	-Higher neighbourhood level socioeconomic status and higher income each significantly increased the likelihood of using hospice -Race has a more powerful effect on hospice use than socioeconomic status neighbourhood -Higher education was associated with a significantly decreased risk of hospitalization at the end of life	10
Khosla et al. (2016) [52]	To investigate the association between socioeconomic status (i.e., education and household income) and anticipatory care planning (ACP) over time using data from the Health and Retirement Study (HRS), a nationally representative survey of middle-aged and older adults living in the contiguous United States	Health and Retirement Study (HRS) (US)	Spouses, partners or proxy informants of HRS respondents (*n* = 6052)	Quantitative Survey to participants	-Very limited support was found for the hypothesis that socioeconomic status would predict ACP -Higher household income increased the odds of having a legally designated power of attorney for health care but had no impact on end of life discussions or written instructions -Education level did not predict engagement in ACP behaviours	10
Lee et al. (2016) [53]	To understand the association of minority race/ethnicity and education with family ratings of the quality of dying and death	Hospital Intensive Care Units (US)	Families of patients who died (*n* = 1290)	Quantitative Survey to participants	-Education was not significantly associated with ratings on the quality of death questionnaire for either patient or family models	11
Schou-Andersen et al. (2016) [54]	To investigate whether demographic and socio-economic factors were associated with preference for dying at home	Community (Denmark)	Relatives of adults who died from cancer (*n* = 282)	Quantitative Survey to participants, database analysis	-At the beginning of palliative period, low income patients more likely to prefer dying at home than high income patients, however medium income patients less likely -At the end of the palliative period, no association between preference for dying at home and income	11
Huang et al. (2015) [45]	To examine the association of individual income and end-of-life care in older cancer decedents in Taiwan	Population-based (Taiwan)	Adults aged over 65 years with cancer (*n* = 28,978)	Quantitative Database analysis	-Low income was associated with more aggressive end of life care -Older cancer decedents with low income were more likely to stay in hospital < 14 days and die in acute hospital -Older cancer decedents with moderate/high income were more likely to have visited the emergency department and admitted to intensive care more frequently	10
Neergaard et al. (2015) [55]	To analyse associations between GP contacts at the end of life and socioeconomic and cultural characteristics of Danish cancer patients	Community (Denmark)	Relatives of adults who died from cancer (*n* = 584)	Quantitative Secondary analysis of pre-existing dataset	-GP face-to-face appointments were higher for decedents with cancer who had low income than those with normal/high income	11
Tang et al. (2015) [56]	To describe longitudinal changes in post-traumatic growth (PTG) during the dying process and to identify determinants of PTG among terminally ill cancer patients	Hospital inpatient units (Taiwan)	Terminally ill cancer patients (*n* = 313)	Quantitative Survey to participants	-Patients with at least senior high school education achieved higher post-traumatic growth scores than those with low education	11
Tucker-Seeley et al. (2015) [57]	To investigate the association between financial hardship and intensive care in the last week of life	Cancer Centers, as part of the Coping with Cancer (CwC) study (US)	Caregivers of deceased cohort (*n* = 281)	Quantitative Secondary analysis of pre-existing CwC data	-Patients who reported financial hardship had higher odds of receiving intensive end of life care -No association between educational attainment or health insurance status and aggressive end of life care -When treatment preferences were included in the fully adjusted model, the association between financial hardship and intensive EOLC was slightly attenuated but remained statistically significant	11
Bhatraju et al. (2014) [58]	To evaluate the factors that were associated with palliative care consultation (PCC) utilization in patients who died in an urban municipal public hospital in the United States, and to examine the association of PCC utilization with symptom management and advance directives at the end of life	Hospital – Inpatients (US)	Patients who died in the units (*n* = 378)	Quantitative Retrospective chart review	-Higher level of education was associated with palliative care consultation (PCC) utilisation -Patients who had PCC were more likely to receive opiates in final 72 h of life, but there was no observed difference in benzodiazepine use	9
Chang et al. (2014) [59]	To assess the association between aggressiveness of end-of-life care and socioeconomic status in working-age terminal cancer patients in Taiwan between 2009 and 2011	Population-based (Taiwan)	Adult cancer patients (*n* = 32,800)	Quantitative Database analysis	-High and moderate socioeconomic status patients had: -Lower scores for aggressive end of life care -Less chemotherapy -Fewer emergency department visits -Less intensive care unit admission -Lower rates of dying in acute care hospitals	11
Lewis et al. (2014) [60]	To explore the nature of social capital in a socioeconomically disadvantaged group of palliative care patients and carers, using a social capital questionnaire to guide and frame discussions	Community (Australia)	Patients who were known to the palliative care service (*n* = 22)	Qualitative Individual interviews	-Overall provision of informal care generally by sole caregiver with intermittent family support -Established neighbour and inter-sectoral networks essential for sustaining care and social needs -Did not feel very engaged with community -Limited communication due to cultural differences -Formalised community care support described as overall being somewhat inconsistent and unpredictable -Patients and carers unsure of formal care being provided -Contact with government agencies (welfare support and government housing) described as positive generally	11
Tang et al. (2014) [61]	To investigate the associations between accurate prognostic understanding and end-of-life care preferences, and to identify correlates of accurate prognostic understanding among terminally ill cancer patients	Hospital (Taiwan)	Terminally ill cancer patients (*n* = 2452)	Quantitative Survey to participants	-Patients with at least senior high school education were 1.28 times more likely to accurately know prognosis -Accurate prognostic understanding associated with greater odds of preferring comfort-oriented care and increased preference for hospice care	11
Chang et al. (2013) [62]	To describe how much burden terminal cancer patients and their caregivers had experienced, what support they most needed, and the differences between them	Hospital (South Korea)	Patients with terminal cancer (*n* = 481) and their caregivers (*n* = 381)	Quantitative Survey to participants	-Low level of education predicted satisfaction about overall care	11
Masucci et al. (2013) [63]	To examine the predictors of the propensity and intensity of five main health service categories in the last three months of life for home-based palliative-care patients	Community. Home-based (Canada)	Family caregivers of patients with malignant neoplasm (*n* = 109)	Quantitative Individual interviews, database analysis	-Patients in the highest deprivation group had higher intensity of home-based nurse visits	11
Sahin et al. (2013) [64]	To evaluate the relationship between different demographic variables and hopelessness, depression and social support in end of life Turkish cancer patients	Hospital (Turkey)	Patients with cancer (*n* = 216)	Quantitative Survey to participants	-Patients with lower education had higher hopelessness scores, but not depression or social support	11
Bergman et al. (2010) [65]	To assess the quality of end of life care in low income uninsured men prospectively enrolled in a specific public assistance program	Population-based (US)	All low-income, uninsured men in the program (*n* = 60)	Quantitative Retrospective chart review, database analysis	-No patients had chemotherapy initiated within 3 months of death, and only 6% had chemo within 2 weeks of death -Use of hospital resources (emergency department visits, inpatient admissions, intensive care) was uniformly low -Hospice utilisation was comparable and timing of referral was better than population	8
Fergus et al. (2010) [66]	To identify key challenges and improvements to out-of-hours palliative care in a mixed urban and rural deprived area	Community (United Kingdom)	Mixed; Patients (*n* = 6), carers (*n* = 1) and healthcare professionals [29]	Mixed Individual interviews, database analysis, observation	-Patients voiced reluctance to use Out of hours primary care phoneline due to: -Stressful and cumbersome process of making initial contact -Bad experiences previously -Misunderstanding of its function -Reluctance to speak to an unknown person -Had bad experiences previously -Did not realise had ‘special notes’ so assumed would be too complex	11
Maric et al. (2010) [67]	To estimate the frequency of anxiety and depression symptoms in patients with advanced lung cancer, and the associations of these with demographic, socioeconomic and clinical factors	Hospital (Serbia)	Patients with stage 3B and 4 non-small cell lung cancer (*n* = 100)	Quantitative Survey to participants	-No difference in levels of psychological distress between education groups or rural versus urban groups -However, unemployed patients had significantly less anxiety and depressive symptoms	9
Chochinov et al. (2009) [68]	To use the Patient Dignity Inventory (PDI), a novel, reliable and validated measure of end-of-life distress, to describe a broad landscape of distress in patients who are terminally ill	Population-based (Canada)	Patients in this program who received palliative care (*n* = 253)	Quantitative Survey to participants	-Patients who were more educated were significantly more likely to report feelings of having lost control, feelings of unfinished business and not being able to perform tasks of daily living	10
Carr et al. (2016) [69]	To explore the extent to which socioeconomic status indicators are associated with attributes considered essential to the quality of one's death	Population-based (US)	Mixed; Bereaved spouses (*n* = 408), patients (*n* = 5276)	Quantitative Secondary analysis of pre-existing datasets	-Limited evidence that socioeconomic status affects death quality, except in pain at the end of life -Wealthier participants were less likely to report severe pain at the end of life, and are more likely to engage in advanced care planning	10
Adler et al. (2019) [70]	To explore the lived experiences of medically underserved women with advanced breast cancer	Community – clinic (US)	Patients with breast cancer and annual family income below 200% of the federal poverty level (*n* = 63)	Qualitative Individual interviews	-Consistent issue of financial distress at the end of life, including difficulty meeting basic needs, inadequate financial resources, inability to work -Patients had concerns about not being “useful” when unemployed and being financially burdensome -Perceived bias in healthcare – some patients felt they were not treated as well by healthcare professionals -Patients viewed fewer financial means, lack of access to private insurance and cancer centres, and minority status as barriers to high quality care -They remained highly attentive to others’ needs and worked to maintain caretaking roles despite own struggles. They made effort to attend to practical matters -Many downplayed desires to discuss dying due to pressure of staying positive -They appreciated meaningful aspects of life (social relationships, creative outlets, spirituality) and felt altruism was important before and during illness	10
Bijnsdorp et al. (2019) [71]	To identify different types of home-based care networks of community dwelling older adults in the Netherlands, and to assess the association between the different types of home based care networks and the health status and sociodemographic characteristics of care recipients	Community (Netherlands)	Respondents who died within 12 months of their last interview, lived at home, and received personal and/or household care (*n* = 146)	Quantitative Survey to participants	-Those with higher education were more likely to have informal care provided by partners whereas those with lower educational attainment more likely to require formal care	11
Saphire et al. (2020) [72]	To examine patterns of symptom management at end of life for older adults who died of lung cancer	Population-based (US)	Individuals who had at least one day in the outpatient non-hospice setting during the last month before death (*n* = 16,246),	Quantitative Database analysis	-High poverty levels were associated with increased receipt of pain, dyspnoea and emotional distress medication	10
Yi et al. (2020) [73]	To compare health and social care costs, quality and their drivers in the last 3 months of life for older adults across countries	Hospital and Community (UK, Ireland, US)	Carers of patients who had accessed a participating palliative care team (*n* = 767)	Quantitative Survey to participants	-Only 4% reported difficult/very difficult financial circumstances, 10% reported just about alright -Higher care costs if “difficulty living on current income” -Hospital care accounts for over 80% total health and social costs, community and palliative costs were low	11
Jacob et al. (2019) [74]	To assess health related quality of life (HRQoL) of advanced cancer patients in terms of general wellbeing (physical, functional, emotional, and social/family wellbeing), pain experiences, psychological state, and spiritual wellbeing, and determine the relationship between belonging to a disadvantaged group and HRQoL outcomes	Hospital (India)	Patients with cancer (*n* = 210)	Quantitative Survey to participants	-Patients with higher financial difficulty scores reported lower functional wellbeing, lower emotional wellbeing, lower meaning/peace (subscale of spiritual wellbeing), and higher anxiety and depression scores. Level of education did not affect this -Even in setting where cancer treatment is free, those with higher financial difficulty scores report lower quality of life outcomes -Scores of this population generally lower than patients in higher income countries e.g., USA	11
Leng et al. (2019) [75]	To explore the prevalence, determinants and consequences of catastrophic health expenditure (CHE) among urban and rural end-of-life cancer patients in China	Community (China)	Families of deceased cancer patients (*n* = 792)	Quantitative Face to face interviews	-There were very high levels of catastrophic healthcare expenditure (CHE) at end of life throughout -1/3 of patients borrowed money from family and friends -Households with higher income were less likely to incur CHE -Rural, low income patients were least likely to access healthcare, inpatient care, and most likely to only use outpatient care. Use of inpatient and outpatient services in these patients increased the risk of CHE -Rural patients allocated higher proportion of household income to healthcare and associated expenditures	11
Saeed et al. (2019) [76]	To study the effect of income and education on the completion of advanced directives	Community and hospital (US)	Patients with cancer (*n* = 265)	Quantitative Secondary analysis of pre-existing VOICE dataset	-Nearly a third of lower income patients had not completed advanced directives (AD) -Patients with lower income had lower AD scores, but there was no significant association between education or perceived financial strain and AD scores	11
Stajduhar et al. (2019) [77]	To identify barriers to assessing care among structurally vulnerable people at the end of life	Community (Canada)	Mixed; People experiencing structural vulnerability in a Western Canadian province (*n* = 25), their support persons (*n* = 25) and formal service providers (*n* = 69)	Qualitative Repeated participant observation, individual interviews	-Structural vulnerability was defined in this study as people living in poverty, in unstable housing, while experiencing various forms of oppression and stigma, e.g., racism, ongoing or past trauma and violence, social isolation, mental and cognitive issues, behavioural issues, substance use, interactions with the criminal justice system, or physical disability -These patients were found to have significant barriers in having their palliative needs met including: -Survival imperative – focussing on basic care needs; -Normalization of dying – often had been told they would die due to addictions -Problem of identification – often not signposted to appropriate health, social benefits -Professional risk and safety management – due to stigmatization from health care professionals -Cracks of a silo-ed care system – complexity of the care systems made them difficult to navigate	10
Wales et al. (2020) [78]	To examine the association between socioeconomic status and other demographic factors on place of death in a population receiving home palliative care in Toronto, Canada	Community—Home palliative care service (Canada)	Patients who died with home-based palliative care service (*n* = 2066)	Quantitative Retrospective chart review	-Rate of preference for home death was significantly lower in lowest income quintile	11

Most studies originated in North America (23/40); 8 in Europe (one of these was UK based); 7 in Asia; 1 Australia and 1 was cross-continent between USA and Europe. The studies were conducted in different healthcare settings including community, hospital and hospice. Some [9] were population studies. Most studies (28/40) recruited patients with cancer diagnoses; 11 did not specify a particular diagnosis and only one looked exclusively at patients with a non-malignant diagnosis – Chronic Obstructive Pulmonary Disease (COPD). Quantitative designs were most commonly used (34/40). There were 3 qualitative studies and the remaining 3 using mixed methodologies. The total sample size of the included studies was 439,423 (range = 18 – 307,188).

The studies included used a variety of definitions to define SED. Twenty-two studies used only one marker in their analysis: education status (*n* = 7); household income (*n* = 4); insurance status (*n* = 3 from the USA); subgroups of employment (*n* = 2); specifically designed SED scores as the single marker of SED (*n* = 2) i.e. the Carstairs SED score and the Index of Relative Disadvantage; decedent postal code linked to nationally available data on poverty rates and estimated income (*n* = 2); patient self-reported financial strain (*n* = 1); structurally vulnerable (*n* = 1). The remaining 18 studies used multifactorial markers of SED.

**Fig. 2:**
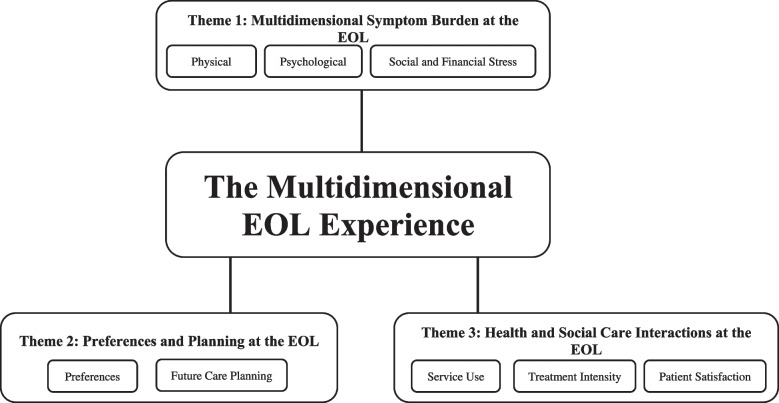
The multidimensional EOL experience: Global themes and subthemes

The amalgamated findings of our research are presented below in 3 global themes (Fig. 2):

- *Multidimensional Symptom Burden at the EOL:* This encapsulates the broad physical, psychological, social and financial issues that patients experience in the last year of life under the subthemes of: physical symptoms, psychological symptoms and social and financial stress.
- *Preferences and Planning at the EOL:* This describes the patient-reported favoured modalities of care in the last year of life and activities taken to plan for such outcomes.
- *Health and Social Care Interactions at the EOL:* This shows how patients use different healthcare services and what their reported satisfaction is with such encounters.

#### Multidimensional symptom burden at the EOL

##### Physical symptoms

There were 3 studies exploring physical symptom burden [42, 44, 69, 72].Lower income and education levels were generally associated with higher physical symptom burden, with pain being widely reported [42, 69]. Poverty was also found to be associated with increased receipt of medication for pain, dyspnoea and emotional distress [72]. However, the association between higher physical symptom burden and SED was not sustained in one study [44] where no difference was found in symptom burden between insured and uninsured patients.

##### Psychological symptoms

The evidence on the relationship between SED and psychological wellbeing is conflicting and was described across 6 of the included studies [50, 56, 64, 67, 68, 74]. While one study found that financial difficulty was associated with lower functional, emotional and spiritual wellbeing [74] another found that unemployed patients with cancer had less anxiety and depression [67]. Similarly, Dhingra et al. found that patients living in impoverished neighbourhoods reported very low illness burden (defined as physical functioning, symptom distress, unmet needs and quality of life) [50]. One study showed that lower education was associated with higher hopelessness and depression rates [64] but education levels generally did not impact on levels of psychological distress, anxiety or depression scores [67, 74].

There is no consistent evidence regarding coping ability. Tang et al. showed that higher education was associated with higher post-traumatic growth scores, showing positive personal development and adjustment to trauma [56]. However, Chochinov et al. reported that patients with higher education were more likely to report feelings of having lost control, unfinished business, and poorer coping with activities of daily living [68].

##### Social and financial stress

Only 2 studies described the social support available to patients, from government agencies and more informal routes [60, 70]. Lewis et al. described limited family support, often a sole caregiver, and sometimes fragile relationships in situations that could be compounded by violence and alcohol in their studied population in a lower socioeconomic area in Western Sydney, Australia. There was a heavy reliance on other sources of support, but welfare support and government housing agencies were described as difficult to navigate and community support (including informal support from neighbours and formalised primary care and community nursing support) was described as inconsistent, unpredictable and inadequate to meet patient need [60]. Once a connection was able to be established, patients who had previously engaged with government agencies prior to their illness described subsequent interactions as a positive experience. However, for those who had no prior connection, the experience, particularly around negotiating benefits was challenging [60].

In one study, women with breast cancer in the US expressed a desire to maintain social connections, describing this as a key aspect of a meaningful life, alongside creative outlets and spirituality. For those living alone, in lower socioeconomic populations, neighbourhood networks were essential to sustain care and social needs [70].

Four studies in countries with both publicly funded and private healthcare systems described the impact of ill-health on finances when living with pre-existing poverty [40, 70, 73, 75]. Financial distress was common prior to diagnosis but worsened after due to medical costs and inability to work. Patients who had low income or who were in financial poverty allocated a larger proportion of family income to health expenses and were more likely to report catastrophic health expenditure and higher health costs at the EOL [73, 75].

Similarly, a qualitative study of low-income patients with breast cancer in America found that most patients had difficulty meeting basic needs, inadequate financial resources, and were unable to work, resulting in them feeling burdensome and useless [70].

Drug reimbursement due to terminal illness (DRTI) is a scheme available in Denmark for patients with incurable disease and a short life expectancy allowing for prescription medicines to be obtained free of charge. However, the authors noted that patients with lower income were less likely to use the DRTI scheme [40].

#### Preferences and planning at the EOL

##### Preferences

Preferences for EOL care were described in 5 studies [49, 54, 61, 76, 78]. Overall, patients with higher education level were more likely to choose supportive care. Tang et al. reported that patients with higher education level were more likely to accurately know their prognosis, which was in turn associated with greater odds of preferring comfort-orientated and hospice-based care at the EOL [61]. Carlucci et al. provided patients with advanced COPD theoretical scenarios and found those with a higher education level were more likely not to choose EOL sustaining treatments such as intubation and non-invasive ventilation. Of note, the study also suggested that all participants’ understanding of choices were suboptimal, with over 40% of participants being unable to correctly define the comfort/supportive option [49]. Saeed et al. found that education did not affect participants’ preference to receive comfort/supportive care [76], however the sample consisted of mainly highly educated participants.

##### Future care planning

Future care planning, including designation of power of attorney, EOL discussions, and completion of written instructions for care at EOL, was described in 3 studies [52, 69, 76]. Khosla et al. found that although higher household income increased the odds of having a legally designated power of attorney for healthcare, this did not impact on EOL discussions or written instructions. They also showed that education level did not impact advanced care planning behaviour [52]. Carr et al. found that having assets significantly increased the likelihood of participants having a living will or legal power of attorney but did not impact on informal EOL discussions [69]. In contrast, another study looking at a sample of lower income patients found that education level and financial strain did not affect completion of advanced care directives [76].

#### Health and social care interactions at EOL

##### Service use

Nine studies described what and how palliative care services were used [40–42, 46, 51, 55, 58, 63, 71]. The evidence shows that even when patients had equal access to palliative care services, differences in the *uptake* of these persisted across different indicators of socioeconomic status. For example, those with higher income or living in a neighbourhood with a higher socioeconomic status, were significantly more likely to have inpatient hospice admissions [51].

The literature points to a distinction between which services are desired or used by patients based on their socioeconomic status. Ankuda et al. showed that patients with financial strain described the connection to social services offered by an at home palliative care service including transportation, help with navigating insurance policies or benefits and food stamps as what mattered most due to self-perceived poverty, disability and high medication cost [41]. Another reason for reliance on practical or social support may be explained by the availability of informal caregivers. For example, Bijnsdorp et al. described a positive correlation between educational attainment and availability of partner care-networks, defined as care provided primarily by a partner in the last year of life, particularly for patients younger than 77.9 years [71]. Similarly, a home-based palliative care service in Canada found a difference in unpaid caregiving hours provided for those with the highest socioeconomic status and the lowest socioeconomic status of 6.18 h and 2.66 h, respectively [46].

When those experiencing SED have accessed specialist palliative care services, there is some evidence that they use them more thereafter. The literature shows that this is the case for both routine and non-routine care and across the different services offered by palliative care. For example, patients with either limited insurance (Medicaid) or no health insurance had more follow up appointments with a hospital palliative care service [42]. This finding persisted for universal, publicly funded health insurance systems. Two Canadian home-based palliative care services showed that patients with higher levels of SED and lower levels of educational attainment had increased propensity and intensity of support worker and nurse visits [46, 63]. The type of support from General Practitioners may also show some slight variation for patients in the last year of life. For example, patients with cancer in the last year of life were more likely to have GP face-to-face visits if they had a lower income [55].

##### Treatment intensity

Treatment intensity at EOL was described in 8 studies [43, 45, 47, 48, 51, 57, 59, 65]. In most of the studies included, patients with lower income, lower education levels, or no insurance, were generally more likely to receive intensive treatment at the EOL [43, 45, 57, 59]. Such treatments included chemotherapy, attendance at emergency departments or > 14 days hospitalisation prior to death, intensive care admission, use of mechanical ventilation and cardiopulmonary resuscitation. Lower income and lower education level also led to increased rate of transfer from home or nursing home to secondary care in the last months of life [48]. Having an inpatient hospice admission during the last 6 months of life reduced hospital admissions by almost half. However, patients with lower income and lower education were less likely to utilise hospice [51].

A few studies were contradictory. A Taiwanese study showed that whilst low income was associated with increased likelihood of hospital admission beyond 14 days and death in an acute hospital, higher income patients were more likely to attend the emergency department and be admitted to intensive care units [45]. One study of cancer patients showed that those with higher education levels were more likely to receive diagnostic and therapeutic procedures at the EOL [47]. Bergman et al. described an analysis of a dedicated programme for men with low income and prostate cancer, and found rates of chemotherapy use, emergency and intensive care admissions and inpatient stays for those in the programme were comparable to the general population [65].

##### Patient satisfaction

There is limited and conflicting evidence relating socioeconomic status to patient satisfaction with palliative care services as described in six of the included studies [41, 53, 62, 66, 70, 77]. Lower levels of education were linked in one study to higher satisfaction with care [62] whilst another showed no association with ratings on quality of death and dying for patients or family [79]. Patients with financial strain rated a home palliative care programme the highest of all socioeconomic groups [41].

The available qualitative research pointed to concerns from patients experiencing SED around the use of palliative care services. For example, patients described that having reduced financial means and lack of private insurance meant they did not receive the same high quality care [79]. Patients living in poverty felt a negative bias and stigmatization from healthcare professionals towards them due to their lower socioeconomic class and this impacted on perception of the care received [79]. One general practitioner out-of-hours service in a deprived area in Scotland was found to be stressful and cumbersome to use with patients describing bad experiences or feeling their care needs were too complex for this service [66]. A Canadian study of those who were homeless, or at risk of such, described that patients often had their care needs unidentified and unmet. However, when patients were linked to palliative care services, they reported feeling listened to and reported that services were extremely accommodating and attentive to their needs [77].

## Discussion

We reviewed, integrated and summarised evidence on the EOL experiences of people living with SED in high income countries. Forty heterogenous studies were identified from a wide range of countries with varying healthcare systems and encompassed both malignant and non-malignant diseases. Three global themes were identified relating to the multi-dimensional EOL experience, preferences and planning and service uptake and utilization. The following key findings were identified—those living with SED have: increased symptom burden; difficulty navigating complex healthcare systems at the EOL and increased intensity of use of these once a link is established; a preference for, and are more likely to have, intensive treatment at the EOL; limited formal and informal social support; a greater propensity to experience financial distress; and, less participation in advanced care planning.

Previous research has shown that people experiencing SED use palliative care services differently and that current universally available models often fail to meet patients’ needs [7, 9–12, 14–18, 40]. Navigating and negotiating multiple and complex systems in order to access essential support may be overwhelming. For example, social service support with transport, navigating insurance, benefits and food stamps mattered most in Ankuda et al. [51], particularly when compounded by unpredictable community support [60, 70] and a lack of unpaid family caregiving [46, 71]. Our work lends further argument to the call for proportionate universalism, where marginalised groups need systems to be designed to incorporate their needs, rather than needing them to adapt to a universal healthcare system, and for time and resources to be proportionate to the level of disadvantage [80, 81].

Dying can be expensive, for both patients and the people caring for them. In addition to existing financial constraint, further loss of income, changes to benefits and treatment-related costs can be catastrophic [70, 75, 82]. Interestingly, even when a drug reimbursement system was available in Denmark, patients from poorer backgrounds were less likely to claim it [40]. This supports what we already know – that financial benefits do not always reach those who need them most. In the UK alone, up to £6.2billion of income-related benefits went unclaimed in 2018–2019 [83]. Ultimately, patients and families may not get access to the financial support they need to mitigate the costs associated with EOL, including fees for diagnostic interventions, hospice or home care and reduced household income as relatives become unemployed unpaid caregivers. This financial distress can leave patients and caregivers feeling devalued, and financially burdensome [70]. Our findings highlight that in order to tackle the systemic issues of social justice which impact on EOL experience, we must adopt multidisciplinary and multi-agency approaches to support families to navigate health and social care and benefits systems. Our review suggests that people experiencing SED valued the non-medical support from palliative care services most, particularly around use of social and supportive services and were less likely to have informal caregivers [46, 60].

Our findings showed that people living in SED absorbed palliative care services when they could gain access to them, with demonstrable higher intensity of use, particularly with regards to outpatient and community-based services [13, 42, 46, 63]. Additionally, our review suggests that patients also have a greater physical symptom burden [42, 69, 84], thus making the greater use of palliative care services unsurprising. However, an alternative explanation could be that services and existing resources are failing to meet the complex needs of people experiencing SED, thus leading to repeated consultations. Barriers to quality care identified in this review have been multifactorial, complex, and difficult to address on a single level. Without significant changes to the way we deliver healthcare, the complexity of these barriers will only serve to widen the quality-of-care gap between deprived and affluent populations yet again, proving the inverse care law true.

Our review demonstrated that people living with SED had both a preference for, and tendency to receive intensive treatments at the EOL [43, 45, 47, 48, 51, 57, 59, 65] and recent research from Scotland added weight to these findings, with Mason et al. demonstrating that for those in the last year of life, living in SED used more unscheduled care (unplanned use of healthcare services) [11]. People living with SED may have more interaction with health and social care professionals and thus potentially more opportunity to engage with future care planning, yet this is not translating to the actual experience at the EOL. Whilst Davies et al. have demonstrated that part of the increased uptake of hospital-based services may be due to poorer health and function for those living in SED [18], until we start asking people about their EOL care experiences and preferences at the right time, in the right way, with consideration given to their limited opportunities to make choices throughout the life course, we will fail to understand the potentially complex nature of future planning for this group.

## Strengths & limitations

This review summarises a thorough analysis of the literature from the developed world about the experience of those dying in SED. We include high quality studies from a variety of settings, healthcare systems and countries thus allowing our work to be transferable and applicable across high income countries. Excluding papers prior to 2010 and addressing well-explored issues of access and preferred place of death has allowed us to focus on the patient voice and experience. In doing so, we acknowledge that this has narrowed the scope of our manuscript but together with the recent reviews on access to palliative care and studies about place of death, there is now a comprehensive and contemporary body of research on how people die and the experiences they have.

Although we included a wide variety of different healthcare settings and systems in various countries, this also meant that there were a wide variety of cultural and societal differences which may limit the generalisability across settings. Indeed the majority of studies (45%, *n* = 18) originated in the United States, which is a similar geographical bias reflected in a previous literature review on access to palliative care services for socioeconomically deprived groups by Lewis et al., 2011 (49%, *n* = 33) [22]. Similar to that group, we acknowledge that the intersection of race with SED and impact of insurance-based health services could have had an impact on our findings. It is significant to note that the magnitude of US research remains persistent ten years later and perhaps reflects differences in availability of funding for palliative care research globally. Whilst we had intended to draw out the patient voice, only 3 studies using a qualitative design and a further 3 studies using mixed quantitative and qualitative research methods. As our findings show, the evidence was heterogenous and often contradictory, which can be challenging to analyse and synthesise. The lack of a standard definition for SED may have contributed to the heterogenous evidence and led researchers of the included papers seeking measures that could be easily obtained.

## Implications for policy, practice and research

The voices of people experiencing SED at the EOL are underrepresented. The COVID-19 pandemic has disproportionately affected people living with poverty [85, 86], forced more families into poverty and has highlighted the fragility of health systems across the globe. Health inequalities are now a stark focus for policymakers globally [87]. Inclusive and participatory research, which enables patients and families to feed directly into this policy making and service design is a priority. We need to actively and broadly engage with policies beyond those of palliative care services at the end of life, to consider the complex needs of this group in societies and systems.

Our review highlighted a dearth of research on spirituality at the EOL for people living with poverty. Further exploration into this important but neglected area is recommended. We also need to understand why people are more likely to experience pain and receive intensive treatment at EOL. In general, patients with lower income were less likely to complete advanced care planning. Further exploration into the link between these two things is important but it is vital that health and social care professionals understand attitudes, experiences and preferences for future care discussions. Further research/implementation science to test specific initiatives in practice is required to better support people living with socio-economic deprivation.

The generalisability of SED research would be improved if standard markers could be agreed upon, at least in similar healthcare systems. Whilst this is not a critique of any particular SED marker, it may be that using a combination of SED markers, or a specifically designed SED score such as the Carstairs SED score would be more appropriate [88]. This echoes the work of social epidemiologists who advocate that valid measurements of socioeconomic status are required and propose the use of multidimensional, composite models which allow for capturing more context [89].

## Conclusion

As a consequence of the COVID-19 pandemic, it is estimated that somewhere between 88 and 115 million people worldwide will be forced into extreme poverty, and issues of inequity have been exacerbated [37]. In the UK, this is happening on the background of a decade of stalling life expectancy, austerity and rising health inequalities between socioeconomic groups and regions [3]. Our comprehensive review shows that SED needs to be a key facilitator in identifying those who are likely to have a greater health burden and thus requiring specialist care at the end of life. Ultimately, future palliative care services cannot adopt a ‘one size fits all’ approach, shaped by our majority populations, rather they should be adaptable and flexible to provide different levels of support based on individualised need. Multidisciplinary and multi-agency approaches are needed to navigate healthcare and benefits systems and tackle the systemic issues associated with socioeconomic deprivation, which impact on EOL experience.

## Data Availability

All data analysed are included in published literature that were identified through the following bibliographic databases: Medline, Embase, CINAHL, ASSIA and PsychInfo. A list of included studies can be found in Table 3. For further information about the availability of these data, please contact the corresponding author.

## Abbreviations

SED: Socio-economic deprivation
EOL: End of life
